# Patterns of regional cerebellar atrophy in genetic frontotemporal dementia

**DOI:** 10.1016/j.nicl.2016.02.008

**Published:** 2016-02-21

**Authors:** Martina Bocchetta, M. Jorge Cardoso, David M. Cash, Sebastien Ourselin, Jason D. Warren, Jonathan D. Rohrer

**Affiliations:** a Dementia Research Centre, Department of Neurodegenerative Disease, UCL Institute of Neurology, Queen Square, London, UK; b Translational Imaging Group, Centre for Medical Image Computing (CMIC), University College London, UK

**Keywords:** Cerebellum, Genetic frontotemporal dementia, *C9orf72*

## Abstract

**Background:**

Frontotemporal dementia (FTD) is a heterogeneous neurodegenerative disorder with a strong genetic component. The cerebellum has not traditionally been felt to be involved in FTD but recent research has suggested a potential role.

**Methods:**

We investigated the volumetry of the cerebellum and its subregions in a cohort of 44 patients with genetic FTD (20 *MAPT*, 7 *GRN*, and 17 *C9orf72* mutation carriers) compared with 18 cognitively normal controls. All groups were matched for age and gender. On volumetric T1-weighted magnetic resonance brain images we used an atlas propagation and label fusion strategy of the Diedrichsen cerebellar atlas to automatically extract subregions including the cerebellar lobules, the vermis and the deep nuclei.

**Results:**

The global cerebellar volume was significantly smaller in *C9orf72* carriers (mean (SD): 99989 (8939) mm^3^) compared with controls (108136 (7407) mm^3^). However, no significant differences were seen in the *MAPT* and *GRN* carriers compared with controls (104191 (6491) mm^3^ and 107883 (6205) mm^3^ respectively). Investigating the individual subregions, *C9orf72* carriers had a significantly lower volume than controls in lobule VIIa-Crus I (15% smaller, p < 0.0005), whilst *MAPT* mutation carriers had a significantly lower vermal volume (10% smaller, p = 0.001) than controls. All cerebellar subregion volumes were preserved in *GRN* carriers compared with controls.

**Conclusion:**

There appears to be a differential pattern of cerebellar atrophy in the major genetic forms of FTD, being relatively spared in *GRN*, localized to the lobule VIIa-Crus I in the superior-posterior region of the cerebellum in *C9orf72*, the area connected via the thalamus to the prefrontal cortex and involved in cognitive function, and localized to the vermis in *MAPT*, the ‘limbic cerebellum’ involved in emotional processing.

## Highlights

• Different pattern of cerebellar atrophy in the major genetic forms of FTD
• Involvement of the lobule VIIa-Crus I in *C9orf72* carriers, related to cognition
• *MAPT* carriers show vermal involvement, linked to emotional processing
• *GRN* carriers show relative sparing of the cerebellum

## 1 Introduction

Frontotemporal dementia (FTD) is a clinically, pathologically and genetically heterogeneous neurodegenerative disorder, commonly presenting with progressive impairment in behaviour (behavioural variant FTD, bvFTD) or language (primary progressive aphasia, PPA). Around a third of patients with FTD have an autosomal dominant mutation in one of three genes: microtubule-associated protein tau (*MAPT*), progranulin (*GRN*) and chromosome 9 open reading frame 72 (*C9orf72*) (Rohrer and Warren, 2011). Neuroimaging and pathological studies of FTD have emphasized the key roles of the frontal, temporal, insular and cingulate cortices as well as subcortical structures such as the striatum and thalamus, but minimal attention has been paid to the potential role of the cerebellum.

Although its function has traditionally been felt to be related solely to the co-ordination of movement, research over the past twenty years has highlighted a role for the cerebellum in cognitive and emotional processing (Schmahmann, 1991, D'Angelo and Casali, 2013, Middleton and Strick, 2000, Strick et al., 2009, Makris et al., 2003). It is extensively connected with different brain regions, including key areas involved in FTD, e.g. via the thalamus to the prefrontal cortex (Behrens et al., 2003, Palesi et al., 2015), and to the limbic system via a direct cerebello-limbic pathway (Arrigo et al., 2014).

Interest in the cerebellum in FTD has arisen from the association of *C9orf72* mutations with cerebellar pathology at *post-mortem* and a number of voxel-based morphometry MRI studies have now found involvement of the cerebellum in this group (Whitwell et al., 2012, Irwin et al., 2013, Mahoney et al., 2012, Rohrer et al., 2015). However detailed region of interest studies have not been performed, nor direct comparison across the different major genetic forms of FTD. Therefore, the aim of this study was to investigate the volume of the cerebellum and its subregions in a cohort of genetic FTD patients, and to determine whether specific cerebellar regions are associated with genetic mutations in *MAPT*, *GRN* and *C9orf72* genes.

## 2 Methods

We reviewed the UCL Dementia Research Centre FTD database to identify all patients who were symptomatic carriers of a mutation in the *MAPT*, *GRN* or *C9orf72* genes and who had also undergone a volumetric T1-weighted MRI. 44 patients were identified: 20 *MAPT* (19 with bvFTD and one with a corticobasal syndrome), 7 *GRN* (3 bvFTD and 4 PPA) and 17 *C9orf72* (13 bvFTD, 2 FTD with motor neurone disease and 2 PPA). No significant differences were seen in age at scan (p = 0.071, Kruskal–Wallis test) or gender (p = 0.301 Chisquare test) between the groups: 57.6 (7.1) years for *MAPT* (75% male), 63.0 (7.0) years for *GRN* (43% male) and 61.4 (6.7) years for *C9orf72* (65% male). Disease duration at time of scan was significantly different between the groups (p = 0.026, Kruskal–Wallis test): 7.0 (4.3) years for *MAPT*, 2.7 (1.9) for *GRN* and 6.0 (4.1) years for *C9orf72*. However, each genetic FTD cause is known to vary in its disease progression and so we examined whole brain volume (corrected for total intracranial volume, TIV) as a proxy of overall disease stage. No significant differences were seen between the groups (p = 0.610, Kruskal–Wallis test): 1108.8 (388.7) cm^3^ for *MAPT*, 1118.9 (411.7) cm^3^ for *GRN* and 1098.1 (661) cm^3^ for *C9orf72*, suggesting that the groups were approximately matched for disease severity. Eighteen cognitively normal subjects, with a similar age and gender to the carriers, were identified as controls: 56.4 (14.3) years (50% male). The study was approved by the local ethics committee and written informed consent was obtained from all participants.

Raw MR images were pre-processed to correct for magnetic field bias (inhomogeneity) using a non-parametric non-uniform intensity normalization (N3) algorithm (Sled et al., 1998, Boyes et al., 2008). We then used an atlas propagation and label fusion strategy of the Diedrichsen cerebellar atlas to automatically extract subregions of the cerebellum: the cerebellar lobules (I-IV, V, VI, VIIa-Crus I, VIIa-Crus II, VIIb, VIIIa, VIIIb, IX and X), the vermis and the deep nuclei (dentate, interposed and fastigial) (Cardoso et al., 2015, Diedrichsen et al., 2009, Diedrichsen et al., 2011). No significant asymmetry was noted in the volumes and so right and left-sided results were combined for each subregion. All volumes were corrected for TIV, which was calculated using the Statistical Parametric Mapping (SPM) 12 software, version 6470 (www.fil.ion.ucl.ac.uk/spm), running under Matlab R2014b (Math Works, Natick, MA, USA) (Malone et al., 2015). Statistical analyses were performed in SPSS software (SPSS Inc., Chicago, IL, USA) version 22.0, with differences in volumes of the cerebellar lobules, vermis and deep nuclei between all groups tested using the Mann–Whitney U test.

## 3 Results

*C9orf72* mutation carriers showed the lowest global cerebellar volume (mean (SD): 99989 (8939) mm^3^), significantly smaller than controls (108136 (7407) mm^3^, p = 0.006 on Mann–Whitney U test), and the *GRN* group (107883 (6205) mm^3^, p = 0.028), but not from the *MAPT* group (104191 (6491) mm^3^; p = 0.080). No significant differences were seen in the *GRN* or *MAPT* groups in comparison with each other or the control group (p = 0.196, *MAPT* versus controls; p = 0.836, *GRN* versus controls; p = 0.370, *GRN* versus *MAPT*).

**Fig. 1:**
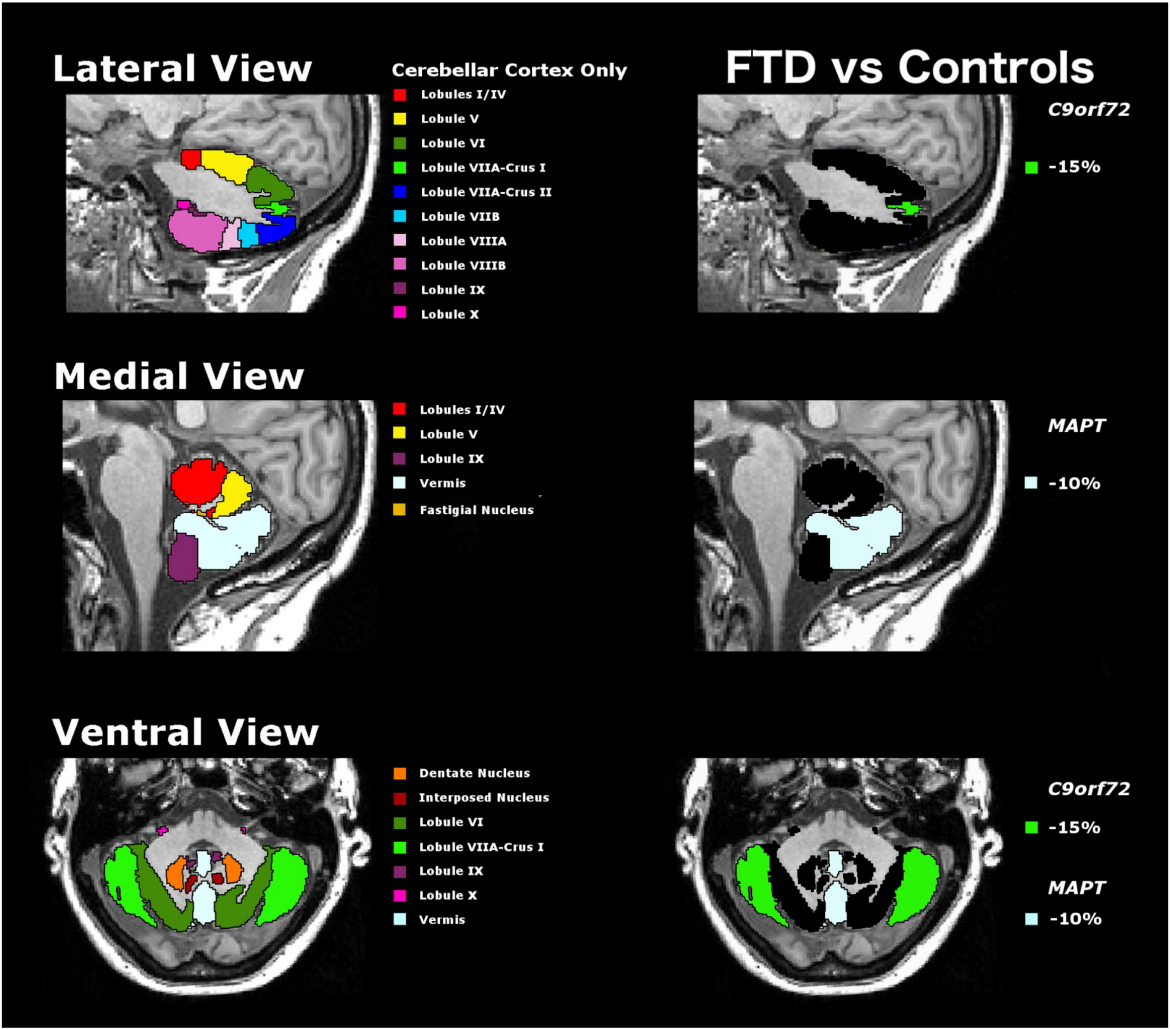
Segmentation of the cerebellar lobules and deep nuclei (left side) mapped on a T1-weighted 3T MR image of a control subject. On the right panel significant volumetric differences in FTD are mapped on the same MR image.

For the 14 individual subregions of the cerebellum, a Bonferroni correction for multiple comparisons was made so that only a threshold of p < 0.003 was considered significant. The *C9orf72* group showed a significantly lower volume compared with controls of the lobule VIIa-Crus I only (p < 0.0005, 15.2% smaller than controls), whilst the *MAPT* group showed a significantly lower volume of the vermis only (p = 0.001, 9.5% smaller than controls) (Table 1 and Fig. 1). No other subregion reached statistical significance but there was a trend for lower volumes of the interposed nuclei in both the *C9orf72* and *MAPT* groups compared with controls (p = 0.004 and p = 0.007, respectively). *GRN* carriers showed preserved cerebellar volumetry with no significant differences from controls.

## 4 Discussion

We found a differential pattern of regional cerebellar involvement in the different genetic forms of FTD: lobule VIIA-Crus I in *C9orf72*, the vermis in *MAPT*, and sparing in the *GRN* group. Lobule VIIa-Crus I has been associated with cognitive processing including the direction of complex goal-directed behaviours, through its connections via the ventrolateral and ventroanterior thalamus to the prefrontal cortex (Makris et al., 2003, Palesi et al., 2015, D'Angelo and Casali, 2013). Although the vermis is involved in the integration of sensory input with motor commands (Bogovic et al., 2013, Schmahmann, 2000, Makris et al., 2003), it is also considered the ‘limbic cerebellum’, involved in the modulation of emotions and social behaviours, based on its connections with the limbic brain structures (Schmahmann, 2000, Stoodley and Schmahmman, 2009, Arrigo et al., 2014, Makris et al., 2003).

The involvement of the cerebellum, and particularly the superior-posterior area, in *C9orf72* mutation carriers is consistent with previous studies (Whitwell et al., 2012, Irwin et al., 2013, Mahoney et al., 2012), but here is localized to a specific lobule (VIIa–Crus I) which is known to be linked to the thalamus, a key area of atrophy in *C9orf72* carriers, and then on to the prefrontal cortex (Behrens et al., 2003, Palesi et al., 2015). Degeneration of a cerebello-thalamic-cortical network has been proposed to underpin disturbances of body schema and related neuropsychiatric symptoms in this group (Downey et al., 2014), and is an early (presymptomatic) feature of the disease (Rohrer et al., 2015).

Identification of vermal involvement in *MAPT* carriers in this region of interest study is unsurprising given what is known about the pattern of atrophy in this group, where limbic structures are predominantly involved, particularly the amygdala, hippocampus and hypothalamus, which are linked to the cerebellum via the cerebello-limbic pathway (Rohrer et al., 2010, Bocchetta et al., 2015). Patients with *MAPT* mutations commonly present with impaired social conduct and behaviour, and it is likely that degeneration across the limbic network leads directly to these symptoms.

There is a clear trend to involvement of the interposed nuclei in both *C9orf72* and *MAPT* groups. As part of the deep cerebellar nuclei, they receive intrinsic inputs from the cerebellar cortex to be sent to the other cortical regions via the ventroanterior and ventrolateral thalamic nuclei (Granziera et al., 2009, Makris et al., 2003), and are likely to be part of the cerebello-thalamic-cortical network in *C9orf72* carriers and the cerebello-limbic network in *MAPT* carriers.

In summary, we found that mutations in *MAPT* and *C9orf72* have a differential impact on the cerebellum with *GRN* carriers having no significant impact. The regions involved are part of different brain networks that are intrinsically linked to the development of the symptoms seen in each of these genetic disorders. It will be important to investigate how early these regions may become affected using data from presymptomatic FTD cohorts such as GENFI (Rohrer et al., 2015). Furthermore, future studies on functional and structural connections of the cerebellum will be helpful in clarifying the role of these networks in genetic FTD.

## Disclosures

JDR is an MRC Clinician Scientist (MR/M008525/1) and has received funding from the NIHR Rare Diseases Translational Research Collaboration (BRC149/NS/MH). JDW is supported by a Wellcome Trust Senior Clinical Fellowship (091673/Z/10/Z). SO is funded by the Engineering and Physical Sciences Research Council (EP/H046410/1, EP/J020990/1, EP/K005278), the Medical Research Council (MR/J01107X/1), the EU-FP7 project VPH-DARE@IT (FP7- ICT-2011-9-601055), and the National Institute for Health Research University College London Hospitals Biomedical Research Centre (NIHR BRC UCLH/UCL High Impact Initiative BW.mn.BRC10269). All other authors have nothing to disclose.

## Figures and Tables

**Table 1:** Volumetry of cerebellar subregions in 18 healthy non-carrier controls and 44 genetic FTD patients. Values denote mean and standard deviation (SD) volumes in mm^3^ or n (%). p-values denote significance on Mann–Whitney U test. Bold represents a significant difference between groups after correcting for multiple comparisons.

	Controls (18)		*C9orf72* (17)		*MAPT* (20)		*GRN* (7)		*C9orf72* vs Controls	*MAPT* vs Controls	*GRN* vs Controls	*C9orf72*	*MAPT*	*GRN*	*GRN* vs *C9orf72*	*GRN* vs *MAPT*	*C9orf72* vs *MAPT*
Structure	Mean	SD	Mean	SD	Mean	SD	Mean	SD	p-Value	p-Value	p-Value	% difference vs controls	% difference vs controls	% difference vs controls	p-Value	p-Value	p-Value
Lobule I–IV	6236	670	6363	861	6250	433	7053	674	0.660	0.534	0.012	− 2.0	− 0.2	− 13.1	0.099	0.013	0.775
Lobule V	8911	897	8623	905	8922	742	9371	737	0.503	0.553	0.125	3.2	− 0.1	− 5.2	0.034	0.145	0.311
Lobule VI	15639	1452	14587	2000	15277	1390	16590	1930	0.067	0.534	0.244	6.7	2.3	− 6.1	0.034	0.104	0.133
Lobule VIIa-Crus I	21091	2467	17885	1984	20360	3303	19298	2237	**< 0.0005**	0.675	0.125	15.2	3.5	8.5	0.099	0.498	0.013
Lobule VIIa-Crus II	17610	2311	15807	2863	16015	1822	17666	752	0.067	0.033	0.534	10.2	9.1	− 0.3	0.147	0.036	0.845
Lobule VIIb	7565	802	7330	976	7488	781	7460	611	0.483	0.515	0.534	3.1	1.0	1.4	0.951	0.850	0.729
Lobule VIIIa	7799	704	7724	927	7957	558	8033	756	0.909	0.361	0.495	1.0	− 2.0	− 3.0	0.534	0.766	0.517
Lobule VIIIb	7451	900	6926	894	7318	1077	7233	834	0.245	0.806	0.790	7.0	1.8	2.9	0.288	0.685	0.270
Lobule IX	6200	828	5563	799	5520	709	5659	864	0.013	0.010	0.158	10.3	11.0	8.7	0.318	0.533	0.988
Lobule X	860	88	850	108	909	124	882	146	0.883	0.228	1.000	1.1	− 5.7	− 2.6	0.757	0.607	0.149
Vermis	5034	375	4624	584	4554	412	4862	408	0.025	**0.001**	0.297	8.1	9.5	3.4	0.260	0.145	0.940
Dentate nuclei	3291	426	3303	406	3214	422	3325	524	1.000	0.361	0.836	− 0.4	2.3	− 1.0	0.951	0.766	0.341
Interposed nuclei	392	44	351	31	357	31	394	54	0.004	0.007	0.836	10.4	8.9	− 0.4	0.099	0.104	0.821
Fastigial nuclei	57	10	51	6	51	6	56	10	0.062	0.033	0.836	10.5	11.1	0.9	0.147	0.104	0.988

## References

[bb0005] Arrigo A., Mormina E., Anastasi G.P., Gaeta M., Calamuneri A., Quartarone A., De Salvo S., Bruschetta D., Rizzo G., Trimarchi F., Milardi D. (2014). Constrained spherical deconvolution analysis of the limbic network in human, with emphasis on a direct cerebello-limbic pathway. Front. Hum. Neurosci..

[bb0010] Behrens T.E., Johansen-Berg H., Woolrich M.W., Smith S.M., Wheeler-Kingshott C.A., Boulby P.A., Barker G.J., Sillery E.L., Sheehan K., Ciccarelli O., Thompson A.J., Brady J.M., Matthews P.M. (2003). Non-invasive mapping of connections between human thalamus and cortex using diffusion imaging. Nat. Neurosci..

[bb0015] Bocchetta M., Gordon E., Manning E., Barnes J., Cash D.M., Espak M., Thomas D.L., Modat M., Rossor M.N., Warren J.D., Ourselin S., Frisoni G.B., Rohrer J.D. (2015). Detailed volumetric analysis of the hypothalamus in behavioral variant frontotemporal dementia. J. Neurol..

[bb0020] Bogovic J.A., Jedynak B., Rigg R., Du A., Landman B.A., Prince J.L., Ying S.H. (2013). Approaching expert results using a hierarchical cerebellum parcellation protocol for multiple inexpert human raters. NeuroImage.

[bb0025] Boyes R.G., Gunter J.L., Frost C., Janke A.L., Yeatman T., Hill D.L., Bernstein M.A., Thompson P.M., Weiner M.W., Schuff N., Alexander G.E., Killiany R.J., DeCarli C., Jack C.R., Fox N.C., ADNI Study (2008). Intensity non-uniformity correction using N3 on 3-T scanners with multichannel phased array coils. NeuroImage.

[bb0030] Cardoso M.J., Modat M., Wolz R., Melbourne A., Cash D., Rueckert D., Ourselin S. (2015). Geodesic information flows: spatially-variant graphs and their application to segmentation and fusion. IEEE TMI.

[bb0035] D'Angelo E., Casali S. (2013). Seeking a unified framework for cerebellar function and dysfunction: from circuit operations to cognition. Front. Neural Circuits.

[bb0040] Diedrichsen J., Balsters J.H., Flavell J., Cussans E., Ramnani N. (2009). A probabilistic MR atlas of the human cerebellum. NeuroImage.

[bb0045] Diedrichsen J., Maderwald S., Küper M., Thürling M., Rabe K., Gizewski E.R., Ladd M.E., Timmann D. (2011). Imaging the deep cerebellar nuclei: a probabilistic atlas and normalization procedure. NeuroImage.

[bb0050] Downey L.E., Fletcher P.D., Golden H.L., Mahoney C.J., Agustus J.L., Schott J.M., Rohrer J.D., Beck J., Mead S., Rossor M.N., Crutch S.J., Warren J.D. (2014). Altered body schema processing in frontotemporal dementia with C9ORF72 mutations. J. Neurol. Neurosurg. Psychiatry.

[bb0055] Granziera C., Schmahmann J.D., Hadjikhani N., Meyer H., Meuli R., Wedeen V., Krueger G. (2009). Diffusion spectrum imaging shows the structural basis of functional cerebellar circuits in the human cerebellum in vivo. PLoS One.

[bb0060] Irwin D.J., McMillan C.T., Brettschneider J., Libon D.J., Powers J., Rascovsky K., Toledo J.B., Boller A., Bekisz J., Chandrasekaran K., Wood E.M., Shaw L.M., Woo J.H., Cook P.A., Wolk D.A., Arnold S.E., Van Deerlin V.M., McCluskey L.F., Elman L., Lee V.M., Trojanowski J.Q., Grossman M. (2013). Cognitive decline and reduced survival in C9orf72 expansion frontotemporal degeneration and amyotrophic lateral sclerosis. J. Neurol. Neurosurg. Psychiatry.

[bb0065] Mahoney C.J., Beck J., Rohrer J.D., Lashley T., Mok K., Shakespeare T., Yeatman T., Warrington E.K., Schott J.M., Fox N.C., Rossor M.N., Hardy J., Collinge J., Revesz T., Mead S., Warren J.D. (2012). Frontotemporal dementia with the C9ORF72 hexanucleotide repeat expansion: clinical, neuroanatomical and neuropathological features. Brain.

[bb0070] Makris N., Hodge S.M., Haselgrove C., Kennedy D.N., Dale A., Fischl B., Rosen B.R., Harris G., Caviness V.S., Schmahmann J.D. (2003). Human cerebellum: surface-assisted cortical parcellation and volumetry with magnetic resonance imaging. J. Cogn. Neurosci..

[bb0075] Malone I.B., Leung K.K., Clegg S., Barnes J., Whitwell J.L., Ashburner J., Fox N.C., Ridgway G.R. (2015). Accurate automatic estimation of total intracranial volume: a nuisance variable with less nuisance. NeuroImage.

[bb0080] Middleton F.A., Strick P.L. (2000). Basal ganglia and cerebellar loops: motor and cognitive circuits. Brain Res. Rev..

[bb0085] Palesi F., Tournier J.D., Calamante F., Muhlert N., Castellazzi G., Chard D., D'Angelo E., Wheeler-Kingshott C.A. (2015). Contralateral cerebello-thalamo-cortical pathways with prominent involvement of associative areas in humans in vivo. Brain Struct. Funct..

[bb0090] Rohrer J.D., Warren J.D. (2011). Phenotypic signatures of genetic frontotemporal dementia. Curr. Opin. Neurol..

[bb0095] Rohrer J.D., Ridgway G.R., Modat M., Ourselin S., Mead S., Fox N.C., Rossor M.N., Warren J.D. (2010). Distinct profiles of brain atrophy in frontotemporal lobar degeneration caused by progranulin and tau mutations. NeuroImage.

[bb0100] Rohrer J.D., Nicholas J.M., Cash D.M., van Swieten J., Dopper E., Jiskoot L., van Minkelen R., Rombouts S.A., Cardoso M.J., Clegg S., Espak M., Mead S., Thomas D.L., De Vita E., Masellis M., Black S.E., Freedman M., Keren R., MacIntosh B.J., Rogaeva E., Tang-Wai D., Tartaglia M.C., Laforce R., Tagliavini F., Tiraboschi P., Redaelli V., Prioni S., Grisoli M., Borroni B., Padovani A., Galimberti D., Scarpini E., Arighi A., Fumagalli G., Rowe J.B., Coyle-Gilchrist I., Graff C., Fallström M., Jelic V., Ståhlbom A.K., Andersson C., Thonberg H., Lilius L., Frisoni G.B., Pievani M., Bocchetta M., Benussi L., Ghidoni R., Finger E., Sorbi S., Nacmias B., Lombardi G., Polito C., Warren J.D., Ourselin S., Fox N.C., Rossor M.N. (2015). Presymptomatic cognitive and neuroanatomical changes in genetic frontotemporal dementia in the genetic frontotemporal dementia Initiative (GENFI) study: a cross-sectional analysis. Lancet Neurol..

[bb0105] Schmahmann J.D. (1991). An emerging concept. The cerebellar contribution to higher function. Arch. Neurol..

[bb0110] Schmahmann J.D. (2000). The role of the cerebellum in affect and psychosis. J. Neurolinguistics.

[bb0115] Sled J.G., Zijdenbos A.P., Evans A.C. (1998). A nonparametric method for automatic correction of intensity nonuniformity in MRI data. IEEE Trans. Med. Imaging.

[bb0120] Stoodley C.J., Schmahmman J.D. (2009). Functional topography in the human cerebellum: a meta-analysis if neuroimaging studies. NeuroImage.

[bb0125] Strick P.L., Dum R.P., Fiez J.A. (2009). Cerebellum and nonmotor function. Annu. Rev. Neurosci..

[bb0130] Whitwell J.L., Weigand S.D., Boeve B.F., Senjem M.L., Gunter J.L., DeJesus-Hernandez M., Rutherford N.J., Baker M., Knopman D.S., Wszolek Z.K., Parisi J.E., Dickson D.W., Petersen R.C., Rademakers R., Jack C.R., Josephs K.A. (2012). Neuroimaging signatures of frontotemporal dementia genetics: C9ORF72, tau, progranulin and sporadics. Brain.

